# Obstetrical and Perinatal Outcomes Are Not Associated with Advanced Paternal Age in IVF or ICSI Pregnancies with Autologous Oocytes

**DOI:** 10.3390/biology12091256

**Published:** 2023-09-20

**Authors:** Ana Navarro-Gomezlechon, María Gil Juliá, Rosa María Pacheco-Rendón, Irene Hervás, Laura Mossetti, Rocío Rivera-Egea, Nicolás Garrido

**Affiliations:** 1 IVIRMA Global Research Alliance, IVI Foundation, Instituto de Investigación Sanitaria La Fe (IIS La Fe), Av. Fernando Abril Martorell, 106, Torre A, 46026 Valencia, Spain; maria.gil@ivirma.com (M.G.J.); rosa.pacheco@ivirma.com (R.M.P.-R.); nicolas.garrido@ivirma.com (N.G.); 2 IVIRMA Global Research Alliance, IVIRMA Roma, Via Federico Calabresi, 11, 00169 Roma, Italy; irene.hervas@ivirma.com (I.H.); laura.mossetti@ivirma.com (L.M.); 3 IVIRMA Global Research Alliance, Andrology Laboratory and Sperm Bank, IVIRMA Valencia, Plaza de la Policia Local 3, 46015 Valencia, Spain; rocio.rivera@ivirma.com

**Keywords:** paternal age, ART, IVF, ICSI, obstetrical outcomes, perinatal outcomes, autologous oocytes, autologous sperm, pregnancy, offspring’s health

## Abstract

**Simple Summary:**

In recent years, there has been an evident delay in parenthood increasing the average parental age at conception. Therefore, there has been a growing interest in the study of the association of age with reproductive outcomes. In this regard, it is well known that advanced maternal age is linked with adverse reproductive outcomes; however, the knowledge regarding the possible effect of advanced paternal age is limited. We studied advanced paternal age in in vitro fertilization and intracytoplasmic sperm injection with autologous sperm and autologous oocytes, dividing the population according to paternal age at conception: ≤30, 31–40 and >40. We found longer pregnancies for the fathers aged 31–40 years compared to those of ≤30 years. Our study supports the findings that advanced paternal age is not associated with clinically relevant obstetrical and perinatal outcomes, except for the duration of the pregnancy. This study provides clinicians and patients with more accurate information on the possible effect of paternal age, sending a hopeful message to couples with aged fathers, and addressing the need for future studies.

**Abstract:**

Background: In recent years, there has been an evident delay in childbearing and concerns have been raised about whether this increase in age affects reproductive outcomes. This study aimed to evaluate the effect of paternal age on obstetrical and perinatal outcomes in couples undergoing in vitro fertilization or intracytoplasmic sperm injection using autologous sperm and oocytes. Methods: This retrospective study evaluated obstetrical and perinatal outcomes from 14,125 couples that were arbitrarily divided into three groups according to paternal age at conception: ≤30 (*n* = 1164), 31–40 (*n* = 11,668) and >40 (*n* = 1293). Statistics consisted of a descriptive analysis followed by univariate and multivariate models, using the youngest age group as a reference. Results: The study showed significantly longer pregnancies for the fathers aged 31–40 compared to ≤30 years. However, there were no significant differences for the type of delivery, gestational diabetes, anaemia, hypertension, delivery threat, premature rupture of membranes, preterm birth, very preterm birth, and the neonate’s sex, weight, low birth weight, very low birth weight, length, cranial perimeter, Apgar score and neonatal intensive care unit admission. Conclusion: Despite our promising results for older fathers, as paternal age was not associated with clinically relevant obstetrical and perinatal outcomes, future well-designed studies are necessary as it has been associated with other important disorders.

## 1. Introduction

Infertility is a problem affecting, more or less, 15% of couples at reproductive age [1,2,3]. Both male and female factors are involved in the achievement of a pregnancy and a live birth [1], so there are many factors that could affect reproductive outcomes in natural as well as assisted reproduction conceptions, and research on this is increasingly important. In this regard, the study of the possible association between the age at conception and reproductive outcomes is gaining relevance [4,5,6,7,8,9,10], due to the evident delay in childbearing in recent years leading to an increase in the average maternal and paternal age at conception [11,12]. While the influence of advanced maternal age, considered >35 years, on reproductive outcomes has been extensively studied [13,14,15,16], knowledge regarding the possible influence of advanced paternal age is limited, with less studies on this topic and a difficulty in establishing a cut-off for advanced paternal age, although it is frequently considered the age of 40 [17,18,19,20,21]. It is well known that advanced maternal age considerably reduces fertility (ovarian reserve and oocyte quality) and increases chromosomal aneuploidy (for example, elevating the risk of Down syndrome) [22]. Moreover, it has been associated with an increased risk of obstetrical and perinatal complications, such as caesarean section [23].

The increase in paternal age may affect epigenetic changes, induce de novo mutations, chromosomal abnormalities, and impair telomere length, which could be responsible for the observed effects on reproductive outcomes, such as offspring heath [24,25,26]. Spermatogonial cells are continuously dividing, which increases the possibility of accumulating mutations. Ashapkin et al. highlighted that age can lead to epigenetic changes in sperm (DNA methylation, histone modifications and small non-coding RNAs profiles) that may be responsible of neurodevelopmental disorders in offspring, such as autism spectrum disorders [27]. Furthermore, paternal age drives changes in the male reproductive system affecting the testis, seminal vesicles, prostate, epididymis, reproductive hormones, sexual function, sperm production and quality, sperm DNA damage and DNA fragmentation [4,6,17,28,29,30,31]. Nonetheless, these molecular mechanisms require further research [6,27]. 

Several studies have found that the increase in male age is associated with a decrease in sperm motility, sperm morphology and seminal volume, but with an increase in sperm concentration [4,31,32]. Nonetheless, García-Ferreyra et al., did not find differences in sperm parameters between the studied age groups [33]. A relatively recent systematic review and meta-analysis in autologous oocyte cycles highlighted significantly higher odds of clinical pregnancy and live birth rates if the paternal age was <35 years or <40 years, and significantly lower odds of miscarriage rate if paternal age was <40 years [26]. Du Fossé et al. found that an advanced paternal age was associated with an increased risk of spontaneous miscarriage [18]. Wu et al. noticed that the increase in paternal age negatively influenced the number of high-quality embryos in intracytoplasmic sperm injection (ICSI) cycles, adjusting for maternal age. However, they did not observe an association with other parameters of embryo quality (number of fertilized oocytes, number of zygotes with two pronuclei and number of viable embryos) and with pregnancy results [34]. Additionally, some authors have noticed an increase in embryo chromosomal aneuploidy with advanced paternal age [33,35], while others have not [7,8]. Kasman et al. found an increased embryo aneuploidy rate for older men when women were under 40 years old [35].

Concerning the possible association of advanced paternal age with obstetrical and perinatal outcomes, advanced paternal age has been associated with an increased risk of stillbirth [36], preterm and very preterm birth [37,38], low birth weight [39,40], low Apgar score [41], gestational diabetes and caesarean section [17,30]. A recent study revealed that advanced paternal age (>44 years) increased the risk of congenital anomalies (adjusted odds ratio (AOR) = 1.17, 95% confidence intervals (CI) 1.12–1.21), especially chromosomal anomalies (AOR = 1.59, 95% CI 1.40–1.78), preterm delivery (<37 weeks (AOR = 1.09, 95% CI 1.08–1.10) and <28 weeks (AOR = 1.06, 95% CI 1.03–1.09)), low birth weight (<2500 g (AOR = 1.11, 95% CI 1.10–1.12) and <1500 g (AOR = 1.06, 95% CI 1.04–1.08)) and admission to a neonatal intensive care unit (NICU) (AOR = 1.13, 95% CI 1.12–1.14) in infants compared with a paternal age of 25–34 years, after adjusting for confounding factors [25]. Nonetheless, some studies have not found an association [42,43,44]. Additionally, in another study from our group considering in vitro fertilization (IVF) or ICSI with donated oocytes, we found lower odds of caesarean delivery and longer pregnancies in fathers aged 31–40 or >40 years and lower odds of having a female infant in fathers aged 31–40 years compared to those ≤30 years old, while other outcomes, such as diabetes, hypertension or the neonate’s weight, remained comparable between groups [45]. Interestingly, Wen et al., noticed that advanced paternal age was negatively associated with the lifespan of male offspring in the Ding genealogy after adjusting for maternal age [24]. Furthermore, advanced paternal age has been linked with an increased risk of some diseases developing in the offspring, including down syndrome, autism spectrum disorders [46], schizophrenia [47], bipolar disorders [17,48], several paediatric [49,50] and adult [51] cancers, orofacial clefts (cleft lip and palate) [17,29,51], achondroplasia [36], Apert syndrome [52,53], and congenital heart defects [51,54,55], as well as cardiovascular abnormalities, facial deformities, urogenital abnormalities, and chromosome disorders [56]. 

Taken together, existing evidence suggests that paternal age could be associated with reproductive risks related to pregnancy and offspring health, which could imply a change in reproductive counselling advising on the risks linked to delayed childbearing derived not only from maternal age, but also from paternal age, so both should be considered in family planning. However, due to the contradictory findings among the available literature and inappropriate study designs, further research is necessary to fully clarify this issue and improve reproductive counselling of couples approaching an infertility clinic. In this regard, our study includes a huge sample size, allowing the evaluation of a large population of patients, and its statistical approach considers multivariate models in which maternal age is included, among others, to control its known effect on the study outcome measures. 

The aim of the present study was to evaluate the effect of paternal age on obstetrical and perinatal outcomes in couples undergoing IVF or ICSI using autologous sperm and autologous oocytes in a large population of patients. Advanced paternal age is often accompanied by advanced maternal age, which is known to affect the outcome measures; for this reason, to perform a more realistic evaluation of the effects of paternal age, we considered maternal age in the adjusted analysis. 

## 2. Materials and Methods

### 2.1. Study Design

The present study follows a retrospective, observational, multicentric and cohort design. The study evaluated the reproductive outcomes of 14,125 couples that underwent an IVF or ICSI cycle at a Spanish IVIRMA clinic (January 2008–March 2020) using the father’s own sperm and autologous oocytes, and from which we had information of the clinical follow-up of the pregnancy and delivery. Aetiologies for infertility varied among the couples included in the study (karyotype alteration, endometriosis, low ovarian reserve, maternal age, premature ovarian failure, polycystic ovarian syndrome, teratozoospermia, oligozoospermia, unexplained infertility, etc.).

We excluded cases in which the semen samples were obtained from testicular biopsy or epididymis aspirate, as well as donor sperm, and combined IVF/ICSI cycles. Moreover, we considered only the first delivery of each patient and only singleton deliveries. We included couples that achieved a pregnancy, whether they had a live birth or not. Patients were arbitrarily divided in three groups according to paternal age ≤ 30 (*n* = 1164), 31–40 (*n* = 11,668) and >40 (*n* = 1293). 

### 2.2. Assisted Reproduction Treatment 

Semen samples were obtained from ejaculate and then liquefied for 30 min at 37 °C and 5% CO_2_. Standard semen analysis was performed to evaluate several microscopic (concentration, motility, and morphology) and macroscopic parameters (volume, pH, and viscosity) followed by sperm preparation using density gradients [57], swim-up [58] or washing. 

Controlled ovarian stimulation and endometrial preparation were performed as previously described [59,60]. Oocytes were recovered and decumulated, succeeded by conventional IVF or ICSI as previously described [61]. Subsequently, embryos were cultured and development was evaluated [57]. Some embryos were biopsied for preimplantation genetic testing (PGT) analysis [62]. At last, the embryos were transferred (fresh or frozen) and a clinical follow-up was performed to measure reproductive outcomes. 

### 2.3. Outcome Measures

Relevant clinical outcomes were obtained from the patient’s clinical charts to build the database to analyse the main outcomes of the study. Data were filtered to remove erroneous or incomplete data. The obstetrical outcomes evaluated were the type of delivery (caesarean versus vaginal), gestational diabetes, anaemia, hypertension, delivery threat, premature rupture of membranes (PROM; before week 37), preterm birth (<37 weeks) and very preterm birth (<34 weeks). The neonate’s gestational age, sex, weight, low birth weight (<2500 g), very low birth weight (<1500 g), length, cranial perimeter, Apgar score (1, 5, and 10 min), and NICU admission were the perinatal outcomes considered. 

### 2.4. Statistical Analysis

R (version 4.2.1) was used for the statistical analysis. In all cases, *p* < 0.05 was considered statistically significant.

We reported the mean with 95% CI for continuous variables and the proportion with 95% CI for categorical variables. To determine the association between paternal age and the study outcomes, we first conducted a descriptive analysis, in which ANOVAs were used to compare the continuous variables and Chi-squared tests were employed for the categorical variables. Then, univariate and multivariate models were also performed applying binary logistic regression for the categorical variables and linear regression for the continuous variables to obtain odds ratios (OR) and regression coefficients (RC), as well as adjusted OR and RC (ARC), respectively. The youngest group of age (≤30 years) was used as a reference for the models. In the multivariate analysis, results were adjusted according to maternal age, maternal body mass index (BMI), last endometrial lining thickness, paternal age, fresh sperm sample concentration and progressive motility, insemination technique, cycle type, treatment, oocyte state, gestational age, transfer on day 5, and type of delivery (when appropriate).

## 3. Results

### 3.1. Baseline Characteristics of the Study Population

Our study included a total of 14,125 couples with fathers aged 18–51 years old. The clinical characteristics of the participants including the patients, assisted reproduction treatment (ART), and semen characteristics are presented in Table 1. Maternal age increased with paternal age and the difference between groups was statistically significant (*p* < 0.001); therefore, to control and avoid this confounding factor in our results, we adjusted for maternal age in the multivariate models. 

### 3.2. Paternal Age and Obstetrical Outcomes

Our first analysis found significant differences between the age groups for gestational diabetes, anaemia, delivery treat, preterm birth and type of delivery, but not for hypertension, PROM and very preterm birth. The application of univariate models revealed that the partners of men >30 years old had a significantly increased risk of developing gestational diabetes or of experiencing a delivery by caesarean section, and a significantly decreased risk of having a delivery threat or a preterm birth than the partners of men aged ≤30 years old. Furthermore, for fathers >40 years old, there was a significantly decreased risk of anaemia in the partner compared to fathers ≤30 years old (Table 2). 

Due to the retrospective nature of the study, we also performed multivariate analysis to avoid potential biases in the results derived from the difference in confounding variables between the groups. Specifically, we adjusted for several potential confounders: maternal age and BMI, last endometrial lining thickness, paternal age, fresh sperm sample concentration and progressive motility, insemination technique, cycle type, treatment, oocyte state, gestational age, embryo transfer on day 5, and type of delivery (when appropriate). There were no significant differences for gestational diabetes, anaemia, hypertension, delivery threat, PROM, preterm birth, very preterm birth, and the type of delivery (Table 2).

### 3.3. Paternal Age and Perinatal Outcomes

For perinatal outcomes, only deliveries that ended in a live birth were considered, which were a total of 14,098 (1160 for ≤30 years old; 11,648 for 31–40 years old; and 1290 for >40 years old). Foetal death rate was 0.09% (0.00–0.48), 0.06% (0.02–0.12) and 0.0% (0.00–0.28) for ≤30, 31–40 and >40 year-old groups, respectively. Perinatal death rate was 0.26% (0.05–0.75), 0.11% (0.06–0.19) and 0.23% (0.05–0.68) for the ≤30, 31–40 and >40 year-old groups, respectively. Overall, the death rate was 0.19%.

Significant differences were observed when comparing the age groups for gestational age in days and cranial perimeter, while no significant relation was noticed for infant sex, weight, low birth weight, very low birth weight, length, Apgar score (1, 5 and 10 min) and NICU admission. Using the youngest group of paternal age as a reference (≤30 years old), no significant results were found for all the perinatal outcomes considered except for gestational age and cranial perimeter. In this regard, we discovered slightly longer gestations when the fathers were 31–40 years old and slightly higher infant’s cranial perimeter when the fathers were >40 years old. Remarkably, after multivariate analysis, paternal age still had a significant effect on the duration of the pregnancy for the fathers aged 31–40 compared to ≤30 years old, while no significant associations were found for the rest of perinatal outcomes measured (Table 3).

## 4. Discussion

The effect(s) of paternal age on obstetrical and perinatal outcomes following IVF or ICSI using autologous sperm and oocytes were evaluated in this retrospective study, with the application of an appropriate statistical approach. Additionally, as normally maternal age is positively correlated with paternal age, confirmed by our data, to avoid this confounding factor and to obtain a more realistic evaluation of the effect of paternal age, we adjusted for maternal age in the multivariate models, among other variables. The multivariate models revealed significantly longer pregnancies for the fathers aged 31–40 compared to those ≤30 years old. Nonetheless, there were no significant differences for gestational diabetes, anaemia, hypertension, delivery threat, PROM, preterm birth, very preterm birth, type of delivery, infant sex, weight, low birth weight, very low birth weight, length, cranial perimeter, Apgar score (1, 5 and 10 min) and NICU admission. Therefore, we did not find an association of paternal age with those variables, which we considered more clinically relevant as they were associated with the health of the mother during pregnancy and of the newborn. 

Although gestational diabetes was more frequent in the partners of older men (5.20% for ≤30, 7.96% for 31–40, and 9.36% for >40 years old), with an AOR of 1.22 (0.66–2.25) for 31–40 years old and 1.07 (0.48–2.40) for >40 years old supporting this observation, these findings were not significant. Similarly, the risk of having a caesarean delivery increased when the father was older (34.82% for ≤30, 38.66% for 31–40, and 50.36% for >40 years old), although the AORs of 0.91 (0.74–1.12) for 31–40 years old and 1.02 (0.75–1.39) for >40 years old did not support this observation. Therefore, other variables were probably more associated with this increase in the risk of caesarean section, such as maternal BMI (*p* < 0.001) and age (*p* < 0.001). Interestingly, among the confounding factors we considered in multivariate models, maternal age was found to be significantly associated with increased odds of gestational diabetes and delivery by caesarean section, and with a decreased odds of delivery threat (*p* < 0.05). In addition, gestational age was revealed to be significantly associated with decreased odds for gestational diabetes, PROM, delivery threat, hypertension, delivery by caesarean, low birth weight, very low birth weight and NICU admission, but increased odds of anaemia and female infants, and an increased infant weight, length, cranial perimeter, and Apgar score at 1 and 5 min (*p* < 0.05 in all cases). Lastly, endometrial lining thickness was also statistically significant in the multivariate models of some variables. Overall, considering the results obtained after multivariate analysis, where we accounted for potential confounders, we can conclude that there may be other variables influencing the outcome measures rather than advanced paternal age.

Our study found decreased odds of preterm birth with age, supported by an increased gestational age, while other researchers found an increased risk of preterm and very-preterm birth in older fathers [37,38]. Similarly, no correlation was found for advanced paternal age and Apgar score at 1, 5 and 10 min, in contrast to the study from Sun et al., who found a modest effect [41]. Recently, Bu et al., after adjusting for potential confounders, revealed that advanced paternal age (>44 years) increased the risk of preterm delivery, low birth weight and NICU admission compared with fathers aged 25–34 years [25]. However, we did not find an association. In addition, Chung et al. [40] and Goisis et al. [39] noticed that paternal age was associated with low birth weight, unlike our study, where we did not observe an association of advanced paternal age with birth weight and low birth weight. ART clinics perform treatments with autologous oocytes and donated oocytes. These oocytes are derived from different populations and require specific treatments, supporting the argument that both oocyte populations should be investigated to identify paternal factors. In a similar, recently published study investigating treatments with donated oocytes, we found that in the older groups there were significantly lower odds of caesarean delivery and having a female infant, in contrast to the present study using autologous oocytes. However, the previous and present studies, similarly, revealed that older groups had longer pregnancies and that other obstetrical and perinatal outcomes were comparable between groups [45]. Remarkably, our findings support those from other studies concluding that an advanced paternal age was not a risk factor for perinatal outcomes [42,43,44]. Neurodevelopmental or genetic disorders in offspring were out of the scope of this study; however, their correlation with advanced paternal age has been well established by several authors [53,63,64] and merits further research, to fully understand these associations and the underlying physiopathological processes. 

Advanced paternal age has been addressed in several investigations suggesting an association with some reproductive outcomes, such as reproductive risks related to pregnancy and offspring health [17,30,37,38,39,40,41,45]. Nonetheless, existing evidence is contradictory and not all studies have correlated paternal age with adverse reproductive outcomes, which raises the need to perform further studies to better clarify this issue. This is particularly relevant because nowadays advanced paternal age is not considered a limitation for ART, and it has been difficult to establish an age threshold [17,18,19,20,21]. In fact, the age of the father is rarely regulated in guidelines for ART. The fact that paternal age could be linked with adverse outcomes could imply a change in reproductive counselling advising on the risks related to delayed childbearing derived not only from maternal age, but also from paternal age, and both should be considered in family planning. The professional community should be informed and should raise awareness in the general population, especially when counselling older parents. Therefore, the establishment of a threshold of advanced paternal age, where fertility decreases and reproductive risks increase, could improve clinical decision-making, and the clinical counselling, risk assessment and fertility care of couples approaching an infertility clinic. 

Overall, it seems that the success of ART is much more influenced by maternal age rather than paternal age. However, some studies have significantly correlated advanced paternal age with adverse ART outcomes. In this regard, our study sends a hopeful message to aged fathers that, considering our results, age does not seem to negatively affect the health of the mother during pregnancy, or of the infant. The major strengths of our study are that it included a huge sample size, allowing the evaluation of a large population of patients, and its statistical approach considers multivariate models in which maternal age is included, among others, to control its known effect on the study outcome measures. It must be noted that due to the retrospective nature of this study, there were some biases derived from the clinical practice, there were some missing data (i.e., incomplete patient histories) and there was some filtering of data that limited the sample size. However, statistical power was still achieved by evaluating a nationwide population cohort. Furthermore, 51 years was the oldest paternal age, so overall fathers were not very old. Future well-designed retrospective, prospective and clinical studies are necessary to better elucidate possible correlations and the underlying molecular mechanisms.

## 5. Conclusions

In recent years, there has been an evident delay in childbearing followed by an increased concern on the possible effect of advanced parental age on adverse reproductive outcomes. In this respect, several studies have shown a correlation of advanced paternal age with the health of the mother during pregnancy and of the infant. Given the importance of the topic, we studied the possible effect of advanced paternal age on obstetrical and perinatal outcomes in IVF or ICSI with autologous sperm and autologous oocytes, finding significantly longer pregnancies for fathers aged 31–40 compared to ≤30 years old. In our study, paternal age was not a risk factor for those more clinically relevant variables, which sends a hopeful message to aged fathers. Nonetheless, these findings must be taken with caution as paternal age has been associated with other important disorders out of the scope of this study. Therefore, future well-designed studies are necessary to better elucidate possible correlations and the underlying molecular mechanisms, with the final goal of improving the reproductive counselling and fertility care of couples approaching an infertility clinic. 

## Figures and Tables

**Table 1:** Baseline characteristics of the patients, assisted reproduction treatment and semen samples for each paternal age group.

	≤30	31–40	>40	*p*
Number of patients	1164	11,668	1293	
Paternal age (years)	28.46 (28.35–28.57)	35.82 (35.77–35.87)	41.75 (41.70–41.81)	<0.001 *
Paternal BMI (kg/m^2^)	23.45 (23.20–23.71)	22.88 (22.81–22.95)	23.19 (22.98–23.40)	<0.001 *
Maternal age (years)	28.22 (28.10–28.34)	35.48 (35.43–35.53)	41.32 (41.26–41.39)	<0.001 *
Maternal BMI (kg/m^2^)	23.38 (23.13–23.62)	22.84 (22.77–22.91)	23.13 (22.92–23.33)	<0.001 *
Last endometrial lining thickness	9.62 (9.50–9.74)	9.60 (9.56–9.64)	9.28 (9.17–9.39)	<0.001 *
Sperm concentration (million/mL)	28.16 (26.39–29.93)	36.86 (36.23–37.48)	46.34 (44.33–48.36)	<0.001 *
Progressive sperm motility	30.27 (28.91–31.63)	33.95 (33.54–34.36)	37.67 (36.56–38.78)	<0.001 *
Number of MII oocytes	11.43 (11.07–11.79)	9.40 (9.30–9.50)	8.68 (8.36–9.24)	<0.001 *
Insemination technique				0.014 *
IVF	1.37% (0.79–2.22)	2.38% (2.11–2.68)	3.17% (2.28–4.28)	
ICSI	98.63% (97.78–99.21)	97.62% (97.32–97.89)	96.83% (95.72–97.72)	
Oocyte state				<0.001 *
Fresh	98.59% (97.72–99.19)	95.81% (95.43–96.17)	95.52% (94.24–96.59)	
Vitrified	1.06% (0.55–1.84)	1.47% (1.26–1.71)	0.55% (0.22–1.13)	
Mixed	0.35% (0.10–0.90)	2.71% (2.42–3.03)	3.93% (2.93–5.15)	
Treatment				<0.001 *
Fresh embryo transfer	55.58% (52.68–58.46)	56.07% (55.16–56.97)	41.53% (38.83–44.27)	
Frozen embryo transfer	44.42% (41.54–47.32)	43.93% (43.03–44.84)	58.47% (55.73–61.17)	
Cycle type				<0.001 *
Stimulated	54.36% (51.42–57.27)	54.87% (53.95–55.78)	40.92% (38.21–43.67)	
Natural	15.51% (13.46–17.73)	15.37% (14.71–16.04)	13.87% (12.03–15.89)	
Substituted	30.14% (27.50–32.89)	29.77% (28.93–30.61)	45.21% (42.46–47.98)	
Sperm preparation method				<0.001 *
Density gradient	42.44% (39.58–45.34)	49.73% (48.82–50.65)	54.29% (51.53–57.03)	
Swim-up	42.96% (40.09–45.86)	39.08% (38.19–39.97)	38.36% (35.70–41.07)	
Only washed	9.54% (7.91–11.37)	5.29% (4.89–5.71)	2.94% (2.09–4.01)	
Embryo transfer				<0.001 *
Prior to day 5	40.98% (38.14–43.88)	37.16% (36.28–38.04)	15.53% (13.59–17.62)	
On or after day 5	59.02% (56.12–61.86)	62.84% (61.96–63.72)	84.47% (82.38–86.41)	

Groups were established according to the paternal age at conception (≤30, 31–40 and >40 years old). Results are expressed as a proportion for categorical variables or mean for continuous variables with corresponding 95% confidence intervals (CI) and a *p* value of the comparisons between age groups. BMI, body mass index; MII, metaphase II; IVF, in vitro fertilization; ICSI, intracytoplasmic sperm injection. * *p* < 0.05.

**Table 2:** Obstetrical outcomes associated with paternal age.

	Proportion (95% CI)	*p*	OR (95% CI)	*p*	AOR (95% CI)	Adjusted *p*
Gestational diabetes		0.03 *				
≤30	5.20% (3.42–7.53)		Reference	–	Reference	–
31–40	7.96% (7.28–8.69)		1.58 (1.05–2.37)	0.028 *	1.22 (0.66–2.25)	0.528
>40	9.36% (7.28–11.79)		1.88 (1.18–3.02)	0.009 *	1.07 (0.48–2.38)	0.878
Anaemia		0.025 *				
≤30	12.95% (10.14–16.20)		Reference	–	Reference	–
31–40	12.14% (11.31–13.01)		0.93 (0.71–1.22)	0.595	0.96 (0.64–1.43)	0.826
>40	8.71% (6.70–11.10)		0.64 (0.44–0.93)	0.02 *	0.79 (0.43–1.46)	0.455
Hypertension		0.964				
≤30	4.83% (3.12–7.10)		Reference	–	Reference	–
31–40	4.70% (4.16–5.28)		0.97 (0.63–1–49)	0.894	0.74 (0.38–1.46)	0.387
>40	4.51% (3.08–6.33)		0.93 (0.54–1.61)	0.794	0.48 (0.18–1.27)	0.139
PROM		0.165				
≤30	3.64% (2.17–5.70)		Reference	–	Reference	–
31–40	2.35% (1.98–2.78)		0.64 (0.39–1.05)	0.078	0.52 (0.21–1.31)	0.167
>40	2.07% (1.14–3.45)		0.56 (0.28–1.13)	0.107	0.58 (0.13–2.64)	0.482
Delivery threat		0.002 *				
≤30	8.28% (6.01–11.07)		Reference	–	Reference	–
31–40	5.57% (4.99–6.19)		0.65 (0.47–0.92)	0.014 *	0.91 (0.52–1.58)	0.738
>40	3.51% (2.26–5.18)		0.40 (0.24–0.68)	<0.001 *	0.53 (0.20–1.41)	0.204
Preterm birth		0.023 *				
≤30	8.90% (7.32–10.69)		Reference	–	Reference	–
31–40	7.15% (6.69–7.64)		0.79 (0.64–0.98)	0.03 *	0.85 (0.59–1.22)	0.376
>40	6.07% (4.82–7.51)		0.66 (0.49–0.90)	0.008 *	0.70 (0.39–1.24)	0.223
Very preterm birth		0.21				
≤30	2.42% (1.61–3.48)		Reference		Reference	
31–40	1.70% (1.47–1.95)		0.70 (0.47–1.04)	0.079	0.65 (0.33–1.26)	0.201
>40	1.79% (1.14–2.67)		0.73 (0.42–1.28)	0.277	0.66 (0.23–1.92)	0.444
Delivery by caesarean section ^a^		<0.001 *				
≤30	34.82% (32.03–37.69)		Reference	–	Reference	–
31–40	38.66% (37.76–39.56)		1.18 (1.04–1.34)	0.012 *	0.93 (0.75–1.14)	0.47
>40	50.36% (47.55–53.16)		1.90 (1.61–2.24)	<0.001 *	1.06 (0.78–1.45)	0.713

Groups were established according to paternal age at conception (≤30, 31–40 and >40 years old). Results are expressed as a percentage with 95% CI, an odds ratio (OR) with 95% CI and a *p* value of the comparison, and an adjusted OR (AOR) and adjusted *p* value. The group ≤30 years old was used as reference for the models. PROM, premature rupture of membranes (prior to 37 weeks). * *p* < 0.05. ^a^ Proportion/probability of caesarean section (rather than vaginal) delivery.

**Table 3:** Perinatal outcomes associated with paternal age.

	Proportion/Mean (95% CI)	*p*	OR/RC (95% CI)	*p*	AOR/ARC (95% CI)	Adjusted *p* Value
Gestational age (days)		0.004 *				
≤30	274.14 (273.31–274.97)		Reference	–	Reference	–
31–40	275.41 (275.17–275.65)		1.27 (0.46–2.07)	0.002 *	1.32 (0.03–2.60)	0.045 *
>40	274.82 (274.09–275.55)		0.68 (–0.38–1.74)	0.209	1.19 (–0.73–3.11)	0.224
Sex ^a^		0.967				
≤30	49.01% (46.03–51.99)		Reference	–	Reference	–
31–40	49.02% (48.08–49.95)		1.00 (0.88–1.13)	0.997	0.95 (0.77–1.16)	0.582
>40	49.40% (46.59–52.21)		1.02 (0.86–1.19)	0.849	0.90 (0.67–1.21)	0.488
Birth weight		0.362				
≤30	3228.27 (3192.61–3263.92)		Reference	–	Reference	–
31–40	3213.04 (3201.90–3224.18)		−15.22 (−51.86–21.42)	0.416	−19.94 (−65.91–26.02)	0.395
>40	3235.87 (3201.83–3269.90)		7.60 (−41.33–56.53)	0.761	9.86 (−59.30–79.01)	0.78
Low birth weight		0.299				
≤30	6.18% (4.69–7.97)		Reference	–	Reference	–
31–40	7.37% (6.83–7.94)		1.21 (0.91–1.61)	0.194	1.23 (0.67–2.24)	0.509
>40	6.51% (5.00–8.30)		1.06 (0.72–1.54)	0.775	0.87 (0.35–2.13)	0.757
Very low birth weight		0.564				
≤30	1.12% (0.54–2.06)		Reference	–	Reference	–
31–40	0.90% (0.71–1.12)		0.80 (0.41–1.54)	0.496	0.78 (0.07–8.23)	0.833
>40	1.19% (0.60–2.12)		1.06 (0.45–2.51)	0.89	1.03 (0.03–40.94)	0.986
Length at birth		0.293				
≤30	49.90 (49.70–50.10)		Reference	–	Reference	–
31–40	49.95 (49.89–50.00)		0.05 (−0.14–0.24)	0.614	−0.02 (−0.27–0.22)	0.86
>40	50.07 (49.92–50.22)		0.17 (−0.07–0.42)	0.164	0.11 (−0.25–0.48)	0.54
Cranial Perimeter		0.032 *				
≤30	34.38 (34.15–34.61)		Reference	–	Reference	–
31–40	34.59 (34.53–34.66)		0.22 (−0.04–0.47)	0.101	0.03 (−0.32–0.38)	0.879
>40	34.79 (34.57–35.00)		0.41 (0.10–0.72)	0.01 *	−0.03 (−0.53–0.47)	0.908
Apgar score 1		0.317				
≤30	8.90 (8.80–9.00)		Reference	–	Reference	–
31–40	8.81 (8.78–8.84)		−0.09 (−0.21–0.03)	0.132	0.02 (−0.15–0.18)	0.858
>40	8.81 (8.73–8.89)		−0.09 (−0.24–0.05)	0.21	0.10 (−0.14–0.34)	0.43
Apgar score 5		0.633				
≤30	9.77 (9.72–9.83)		Reference	–	Reference	–
31–40	9.74 (9.72–9.76)		−0.04 (−0.11–0.04)	0.373	−0.03 (−0.14–0.07)	0.534
>40	9.73 (9.68–9.79)		−0.04 (−0.14–0.05)	0.368	−0.02 (−0.17–0.14)	0.825
Apgar score 10		0.971				
≤30	9.86 (9.78–9.93)		Reference	–	Reference	–
31–40	9.85 (9.81–9.88)		−0.01 (−0.12–0.09)	0.824	0.00 (−0.17–0.17)	0.985
>40	9.84 (9.76–9.92)		−0.02 (−0.15–0.12)	0.823	0.00 (−0.25–0.25)	0.995
NICU Admission		0.674				
≤30	7.75% (5.62–10.37)		Reference	–	Reference	–
31–40	6.77% (6.14–7.44)		0.86 (0.62–1.21)	0.392	0.78 (0.44–1.40)	0.411
>40	7.08% (5.36–9.13)		0.91 (0.59–1.38)	0.649	0.58 (0.25–1.35)	0.208

Groups were established according to the paternal age at conception (≤30, 31–40 and >40 years old). Results are presented as a proportion or mean with corresponding 95% CIs and computed *p* values of the comparison between the three groups, the OR or regression coefficient (RC) with corresponding 95% CI and a *p* value of the comparisons, and the AOR or adjusted RC (ARC) and adjusted *p* values. The group ≤30 years old was used as reference for the models. NICU, neonatal intensive care unit. * *p* < 0.05. ^a^ Percentage of live female births over the total number of live births.

## Data Availability

Not available.
